# Follicular fluid thyroid autoantibodies, thyrotropin, free thyroxine levels and assisted reproductive technology outcome

**DOI:** 10.1371/journal.pone.0206652

**Published:** 2018-10-29

**Authors:** Sanja Medenica, Eliana Garalejic, Biljana Arsic, Biljana Medjo, Dragana Bojovic Jovic, Dzihan Abazovic, Rade Vukovic, Milos Zarkovic

**Affiliations:** 1 Department of Endocrinology, Internal Medicine Clinic, Clinical Center of Montenegro, Podgorica, Montenegro; 2 In Vitro Fertilisation Department, Clinic for Gynecology and Obstetrics "Narodni front", Belgrade, Serbia; 3 School of Medicine, University of Belgrade, Belgrade, Serbia; 4 Pediatric Intensive Care Unit, University Children’s Hospital, Belgrade, Serbia; 5 Emergency Medicine Center of Montenegro, Podgorica, Montenegro; 6 Department of Endocrinology, Mother and Child Healthcare Institute of Serbia “Dr VukanCupic”, Belgrade, Serbia; 7 Department of Thyroid Gland Disease, Clinic of Endocrinology, Diabetes and Metabolic Diseases, Clinical Center of Serbia, Belgrade, Serbia; University of Crete, GREECE

## Abstract

**Objective:**

Although there are substantial data linking thyroid autoimmunity (TAI) and infertility, data regarding assisted reproductive technology (ART) outcomes and TAI markers in follicular fluid (FF) of women undergoing ART are scarce. Objective of the study was to assess the association of the levels of thyroid autoantibodies in FF and ART outcome expressed as the achieved pregnancies.

**Methods:**

This study enrolled 52 women undergoing ART (26 TAI positive subjects and 26 age and body mass index matched TAI negative controls). Blood samples were drawn before the initiation of protocol for controlled ovarian stimulation, and thyrotropin (TSH), free triiodothyronine (fT_3_), free thyroxine (fT_4_), thyroid peroxidase antibodies (TPOAbs) and thyroglobulin antibodies (TgAbs) levels were measured. TSH, fT_4_, TPOAbs, TgAbs and progesterone levels were also measured in FF.

**Results:**

There were no significant differences between the groups regarding mean levels of FF TSH and FF fT_4_. Statistically significant correlation was discovered regarding the levels of serum and FF TPOAbs (0,961, p<0.001 in TAI positive, 0,438, p = 0.025 in TAI negative group) and TgAbs (0,945, p<0.001 in TAI positive, 0,554, p = 0.003 in TAI negative group). Pregnancies rates per initiated cycle and per embryotransfer cycle were significantly different between TAI positive and TAI negative group, (30.8% *vs* 61.5%), p = 0.026 and (34.8% *vs* 66.7%), p = 0.029, respectively. Multivariate analysis showed that TAI positive women had less chance to achieve pregnancy (p = 0.004, OR = 0.036, 95% CI 0.004–0.347).

**Conclusions:**

Higher levels of thyroid autoantibodies in FF of TAI positive women are strongly correlated with serum levels and may have effect on the post-implantation embryo development.

## Introduction

Thyroid autoimmunity (TAI) is the most prevalent autoimmune disease in women of reproductive age, affecting 5%-20% of female population [1]. Hashimoto thyroiditis (HT) is the most common clinical presentations of TAI, characterized by the presence of thyroid autoantibodies, including thyroid peroxidase antibodies (TPOAbs) and thyroglobulin antibodies (TgAbs) [2], mediating antibody-dependent cell-mediated cytotoxicity [3–5]. Numerous studies have focused on association of TAI, infertility and obstetrical complications[6–9]. The hypotheses have been suggested to explain possible connection between TAI and obstetrical complications. In the first one, TAI is considered to be a consequence of general autoimmune response, explaining higher rate of ‘fetal graft’ rejection [10]. The second hypothesis implies that TAI could be associated with thyroid hormones deficiency, or inability of thyroid gland to adapt to hormonal changes during pregnancy [11]. TAI is also associated with an increased risk of unexplained subfertility [12]. It was suggested that thyroid autoantibodies may serve as independent markers of assisted reproductive technology (ART) outcome failure [13]. The risk for miscarriage may be higher in euthyroid, subfertile women with TAI undergoing ART [10], with lower pregnancy rate [14] compared with subfertile women without TAI. Controlled ovarian stimulation, as a part of ART procedure, seems to have a long-term impact on TSH levels [15], leading to a significant increase of serum TSH in the very first period of pregnancy and alter thyroid function in euthyroid TAI positive patients [16].

Follicular fluid (FF) provides an important microenvironment for oocytes maturation and development [17]. Monteleone et al, verified the presence of thyroid autoantibodies in FF of TAI positive women, suggesting that these antibodies could cross the follicule-blood barrier and damage maturing oocytes used in ART procedure, due to antibody mediated cytotoxicity [14].

The objective of the study was to assess the association of the levels of thyroid autoantibodies in FF and ART outcome expressed as the achieved pregnancies.

## Subjects and methods

This prospective study was conducted during the period from November 2014 to July 2016, in the Clinic for Gynecology and Obstetrics "Narodni front", Belgrade, Serbia. We enrolled 26 euthyroid subjects with TAI undergoing ART, and 27 euthyroid age and body mass index (BMI) matched TAI negative subjects undergoing ART (one of these subjects has withdrawn consent during the later course of the study).

### Ethical approval

The ethical committee of the Faculty of Medicine, University of Belgrade and the ethical committee of Clinic for Gynecology and Obstetrics "Narodni front", granted approval for the present study and written informed consents were obtained from all subjects.

### Study population criteria

ART procedure inclusion criteria were predefined by the National Expert Commission of the Ministry of Health for biomedical assisted fertilization procedures [18]. Inclusion criteria for ART: couples in whom other possibilities for infertility treatment have been exhausted, women with infertility despite appropriate treatment, women up to age 40 years, with preserved ovarian function, with BMI<30 kg/m^2^, all forms of male subfertility with the presence of live or morphologically correct sperm in the ejaculate. Exclusion criteria for ART: anomalies and benign tumors of the uterus, fallopian tubes and ovaries that disable ART procedure, the achieving and development of pregnancy, the existence of a malignant tumor or suspicious malignancy in the uterus, fallopian tubes and ovaries, any diseases that can significantly influence pregnancy or pregnancy outcome, and diseases in which the anesthesia or pregnancy potentially can threaten the mother’s life.

### Clinical methodology

Medical history, physical examination and laboratory analyses were performed in each patient and demographic, anthropometric data, history of thyroid disease and documentation of ART were collected.

The blood samples were drawn in the morning on the day when the protocol for controlled ovarian stimulation was started. Serum thyrotropin (TSH), free triiodothyronine (fT_3_), free thyroxine (fT_4_), TPOAbs and TgAbs levels were measured by automated analyzer Cobas 6000 ROCHE, using commercial tests of the same company based on the principle of *immuno-chemiluminescence* method.Reference values were: TSH 0.40–4.00 μIU/ml, fT_4_ 12.00–22.00 pmol/l, fT_3_ 3.10–6.80 pmol/l, TgAbs 0.0–25.3 IU/ml, upper limit of TPOAbs reference range 19 IU/ml (95% CI 17–26 IU/ml) adapted for the local population in Serbia [19]. Criterion for euthyroidism was serum TSH in general population reference range.

For controlled ovarian stimulation, we used one of two protocols, according to a personalized regimen, long gonadotropin-releasing hormone agonist (GnRH-a), (Dipherelin 0,1 mg/ml, Triptorelin, Pharma Swiss, Belgrade, Serbia) or short GnRH-antagonist (GnRH-ant), (Cetrotide 0.25 mg/ml, Cetrorelix acetate, Merck Serono, Frankfurt, Germany), in combination with urinary HMG-a (Menopur 75i.j., menotrophin-human menopausal gonadotropin HMG, Ferring Pharmaceuticals, Fering B.V.) and/or a recombinant follicle-stimulating hormone FSH (Gonal-F 75i.j., follitropin alpha, Merck Serono, Modugno, Italy and/or Puregon 50 IU and 100 IU follitropin beta, Merck Sharp & Dohme, Belgrade, Serbia). The initial doses of gonadotropin drugs were adjusted according to the patient age, the estimated ovarian reserve, and according to the response to prior stimulation, and during the first three to four days the initial doses were fixed. Further, transvaginal ultrasonography, together with measuring of blood estradiol (E2), were used to estimate the ovarian response, and adjust the gonadotropin dose. E2 was measured using an automatic imunoanalyzer Elecsys (Roche Diagnostics, Mannheim, Germany), and intra-assay and inter-assay coefficients of variation were 5% and 10%. When 3 or more follicles larger than 17 mm were detected with adequate E2 level, final oocyte maturation by 10 000 IU human horionic gonadotropin hCG (Pregnyl, Chorionic gonadotropin, Merck Sharp & Dohme, Belgrade, Serbia) or Ovitrelle 250 micrograms (choriogonadotropin alfa, Merck Serono SpAModugno, Italy) was done. In the case of poor ovarian response (a small number of growing follicles) final oocyte maturation was induced, if there was only one follicle size 17 mm. The oocyte aspiration was done 35h hours later under the ultrasound control. Intracytoplasmic sperm injection (ICSI) or *in vitro* fertilization (IVF)/ICSI and embryo transfer were preformed, as appropriate. In FF, obtained from follicles ≥ 17 mm, TSH, fT_4_, TPOAbs, TgAbs and progesterone (P4) levels were measured by automated analyzer Cobas 6000 ROCHE, using commercial tests of the same company based on the principle of *immuno-chemiluminescence* method.Progesterone was administered for the luteal support.

### Study population

Final study population included 52 subjects, 26 in TAI positive and 26 in the control TAI negative group. Among the 26 patients of TAI positive group, 20 patients were on levothyroxine substitution. TAI negative group included patients with serum thyroid autoantibodies level within normal range.

### Analyzed data

We analyzed general characteristics of patients undergoing ART and ART related information. Biochemical pregnancy was confirmed by determining the β subunit of hCG> 50 mIU/ml in serum 16 days after oocyte aspiration, clinical pregnancy was determined in the seventh week of gestation (diagnosed by vaginal ultrasound examination, the presence of gestational sac or the heart beat of the fetus), and early miscarriages as spontaneous loss of pregnancy from the seventh to the twelfth week of gestation.

### Statistical analyses

We used G*Power to calculate sample size with the following parameters: two tails, α err prob = 0.05, power = 0.7. Calculated sample size was 52 [20].

Results are presented as mean±sd, median or absolute numbers (%). T test, Mann-Whitney U test and Chi-square test were used to assess significant differences between groups. Spearman correlation analysis was used to evaluate correlation between numerical variables. Variables with non-normal distribution were transformed using logarithmic transformation. Univariate and multivariate logistic regression analysis were used for outcome prediction. In multivariate models, multicollinearity was examined using VIF (variance inflation factor). The calibration of model was tested using the Hosmer-Lemeshow goodness of fit test. All p values less than 0.05 were considered significant.

## Results

Study population consisted of 52 patients. At the time of enrollment, all patients were nullipara, except one which was unipara. All patients had primary infertility, except one patient with secondary infertility. General characteristics of the study group are presented in Table 1.

**Table 1:** General characteristics of study population in regards to the presence of serum thyroid autoantibodies.

			TAI		p value
			Positive	Negative	
Mean age (year)			34.6±3.5 (26–40)	33.9±3.8 (24–40)	0.476
Mean BMI (kg/m^2^)			23.3±2.7	22.3±2.7	0.178
Smoking (%)			19.2	42.3	0.132
Family history for autoimmune diseases (%)			26.9	7.7	0.140
Personal history for other autoimmune diseases, vitiligo or allergic rinitis (%)			15.4	11.5	1.000
Mean infertility duration (months)			51.5±22.0	77.0±50.3	0.050
Previous miscarriage (%)			7.7	11.5	0.638
Previous ART (%)			26.9	34.6	0.548
Antral follicle count			14.35±9.65	10.92±7.06	0.340
AMH (ng/ml)			2.66±1.84	2.16±1.47	0.390
AMH/upper reference range for the age (%)			46.71±37.54	33.10±22.13	0.253
Etiology of infertility(%)	Male infertility		53.8	30.8	0.228
	Female infertility	Endometriosis	3.8	11.5	
		PCOS	7.7	0	
		tubal factor	3.8	11.5	
	Both male and female infertility		7.7	19.2	
	Infertility of unknown cause		23.1	26.9	

TAI-thyroid autoimmunity referring to the presence of serum thyroid autoantibodies (thyroid peroxidase antibodies and/or thyroglobulin antibodies); BMI-body mass index; ART-assisted reproductive technology; AMH-Anti-Müllerian hormone; PCOS-polycystic ovary syndrome

### Characteristics of thyroid disease in patients with positive thyroid autoantibodies

Treatment duration in 20 TAI positive subjects on levothyroxine therapy was 19.50 months (4–134, median, range), mean daily levothyroxine dose was 67.49±29.40 mcg. No statistically significant differences in terms of TSH (1.56±0.70 *vs* 1.90±0.43 µIU/ml, p = 0.324), fT_3_ (4.75±0.51 *vs* 5.39±0.98 pmol/l, p = 0.135), and significant difference regarding fT_4_ (20.98±2.93 *vs* 16.52±3.52 pmol/l, p = 0.006) were found between patients in TAI positive group receiving levothyroxine substitution and those with no treatment, respectively.

### Serum and follicular fluid parameters, ART characteristics and outcomes

All patients who have achieved biochemical, have also achieved clinical pregnancy. Embryo transfer (ET) was not performed in 3 patients in TAI positive, and in 2 patients in TAI negative group.

Table 2 shows levels of serum TSH, fT_4_, fT_3_, TPOAbs, TgAbs, and FF TSH, FF fT_4_, FF TPOAbs, FF TgAbs, and ART characteristics and outcomes in TAI positive and negative groups. Our results showed a significant positive correlation between serum and FF TPOAbs (0.961, p<0.001) and TgAbs (0.945, p<0.001) in TAI positive group, and significant correlation between serum and FF TPOAbs (0.438, p = 0.025) and TgAbs (0.554, p = 0.003) in TAI negative group.

**Table 2:** Serum and follicular fluid parameters, assisted reproductive technology characteristics and outcomes in patients with positive thyroid autoantibodies (TAI positive) and patients with negative thyroid autoantibodies (TAI negative).*

	TAI		p value
	Positive	Negative	
Serum TSH (μIU/ml)	1.63±0.65	2.22±0.89	0.010
Serum fT_4_ (pmol/l)	19.95±3.56	17.76±1.84	0.008
Serum fT_3_ (pmol/l)	4.89±0.68	5.51±0.49	0.001
Serum TPOAbs (IU/ml)	56.50 (5.50–600.00)	11.35 (6.80–25.50)	<0.001
Serum TgAbs (IU/ml)	66.14 (9.00–2380.00)	9.00 (9.00–22.99)	<0.001
FF TSH (μIU/ml)	1.52±0.94	1.54±0.76	0.921
FF fT_4_ (pmol/l)	16.28±2.36	15.70±1.73	0.319
FF TPOAbs (IU/ml)	27.73 (4.00–525.20)	4.00 (4.00–89.80)	<0.001
FF TgAbs (IU/ml)	38.29 (9.00–1488.00)	9.00 (9.00–18.35)	<0.001
Long GnRH-a *vs* short GnRH-ant (%)	38.5 *vs* 61.5	26.9 *vs* 73.1	0.375
Stimulation lenght (days)	10.77±1.31	10.23±1.17	0.125
Total gonadotropin dose (IU)	2414.4±698.2	2567.3±607.3	0.404
Serum E2 on the day of final injection (pmol/l)	7068.7±2808.7	6718.7±3122.2	0.621
ICSI *vs* IVF/ICSI (%)	65.4 *vs* 34.6	73.1 *vs* 26.9	0.548
Pathological spermogram on the day of oocyte retrieval (%)	69.2	80.8	0.337
Number of retrieved oocytes	9.65±4.66	8.42±4.29	0.474
Number of good quality oocytes	6.62±3.42	6.19±3.43	0.869
Percentage of good quality oocytes (%)	69.35±15.46	70.99±20.75	0.748
Fertilization rate (%)	69.57±24.54	66.61±26.25	0.676
Number of embryos	5.15±3.12	4.46±2.83	0.417
Number of top quality embryos	4.63±2.34	4.13±2.27	0.350
Number of embryo transferred	2.35±0.57	2.25±0.53	0.516
2^nd^*vs* 3^rd^ day of ET (%)	73.9 *vs* 26.1	75.0 *vs* 25.0	0.932
Implantation rate (%)	21.01±32.65	31.94±24.53	0.092
Pregnancy rate per initiated cycle (%)	30.8	61.5	0.026
Pregnancy rate per ET cycle (%)	34.8	66.7	0.029
Twin pregnancy rate (%)	25.0	50.0	0.388
Early miscarriage rate (%)	0.0	12.5	0.536

*Results are expressed as mean±SD or %, with median and ranges given for levels of autoantibodies.

TAI-thyroid autoimmunity referring to the presence of serum thyroid autoantibodies (thyroid peroxidase antibodies and/or thyroglobulin antibodies); TSH-thyrotropin, fT_4_-free thyroxine, fT_3_-free triiodothyronine, TPOAbs- thyroperoxidase antibodies; TgAbs-thyroglobulin antibodies; FF-follicular fluid; GnRH-a- gonadotropin-releasing hormone agonist; GnRH-ant- gonadotropin-releasing hormone antagonist; E2-estradiol; hCG-human chorionic gonadotropin; ICSI-intracytoplasmic sperm injection; IVF-in vitro fertilization, ET-embryo transfer

We demonstrated slightly higher level of serum fT_4_ (19.9±3.2 vs 17.7±2.8, p = 0.017), in patients with no pregnancy per ET cycle, with no significant differences regarding TSH (1.9±0.9 *vs* 2.0±0.8, p = 0.536), fT3 (5.0±0.6 *vs* 5.3±0.6, p = 0.117), serum TPOAbs (75.8±122.4 *vs* 56.1±127.6, p = 0.085), serum TgAbs (138.8±305.4 *vs* 217.0±606.4, p = 0.827), FF TSH (1.5±0.9 *vs* 1.7±0.8, p = 0.376), and FF fT4 (16.2±2.0 *vs* 15.8±2.3, p = 0.471), FF TPOAbs (36.0±55.6 *vs* 39.1±108.1, p = 0.356), FF TgAbs (68.1±92.7 *vs* 137.9±377.4, p = 0.683) in patients with no pregnancy per ET cycle.

Different etiologies of infertility (male, female, both male and female, unknown cause) did not have statistically significant impact on ART outcome (pregnancy per ET cycle p = 0.500; pregnancy per initiated cycle p = 0.437).

In univariate logistic regression model with pregnancy per ET cycle as the outcome we showed that the chance for achieving pregnancy is 3.75 times higher in TAI negative women, as opposed to TAI positive women (p = 0.032, OR = 0.267, 95% CI 0.080–0.891).

Multivariate analysis with predictors being TAI (positive /negative), log FF P4, previous ART and long GnRH-a protocol (Table 3) showed that TAI positive women had significantly less chance to achieve pregnancy (p = 0.004, OR = 0.036, 95% CI 0.004–0.347).

**Table 3:** Assisted reproductive technology outcome predictors.

R squared = 0,611				
	P value	OR	95% C.I.for OR	
			Lower	Upper
	TAI positive	.004	.036	.004	.347
	log_P4	.011	56.276	2.542	1245.660
	Previous ART	.013	.077	.010	.578
	Long GnRH-a protocol	.005	50.332	3.235	783.165
	Constant	.017	.000		

TAI-thyroid autoimmunity referring to the presence of serum thyroid autoantibodies (thyroid peroxidase antibodies and/or thyroglobulin antibodies); P4-progesterone; ART-assisted reproductive technology; GnRH-a- gonadotropin-releasing hormone agonist

## Discussion

The present study verified the presence of thyroid autoantibodies in FF of TAI positive women undergoing ART, and assessed their impact on achieving fertility. To the best of our knowledge, for the first time, we demonstrated the presence of TSH and fT_4_ in FF of TAI positive woman undergoing ART, and estimated their impact on the fertilization and embryo development during ART. Our study highlighted that TAI positive women have less chance to achieve pregnancy. The major limitation of our study is the relatively small power of the study. Also, our study included 52 (26+26) patients, while Monteleone study included 31 (14+17) patients.

The only difference between TAI positive and negative group was duration of infertility. Most of the patients in TAI positive group have already been diagnosed with TAI, and treated with levothyroxine. The difference in duration of infertility could be explained by the fact that TAI patients made earlier decision to undergo ART programme.

Our results showed mean serum TSH and fT_3_ levels were higher, but mean serum fT_4_was lower in TAI negative group. It could be explained by the levothyroxine substitution in TAI group to achieve lower TSH before undergoing ART [21–23]. Adequate substitution in TAI patients with and without hypothyroidism reduce miscarriage rate or preterm birth [24,25]. Mature (MII) oocytes from women undergoing ART demonstrate the presence of thyroid hormones receptors, supporting the hypothesis that T_3_ has a direct effect on the human oocytes, indicating a possibility of conversion of peripheral T_4_ on ovarian tissue [26]. T_3_ have a direct impact on oocytes [27,28] and granulose cells in combination with FSH acting as ‘biological amplifier’ of the FSH stimulatory function [29]. One preliminary observational study showed that lower serum fT_3_ levels in women with HT on levothyroxine substitution may contribute to the higher infertility rate [30], as in TAI positive group in our study. In the study of Monteleone et al., no significant difference was found between TAI positive and negative group according to serum TSH, fT_4_ and fT_3_ level [14].

No difference was found between TAI positive and negative group according to the mean TSH and fT_4_ level in FF. Studies show that women with infertility have higher fT_4_ concentrations in FF than healthy population [31].

TAI positive group of patients, as was expected, had significantly higher mean serum and FF TPOAbs and TgAbs levels, in comparation to TAI negative group, with statistically significant correlation between levels in serum and FF in both groups.

No statistically significant difference was found between the groups according other ART characteristics, but high difference was found between TAI positive and negative group in pregnancy rate per initiated cycle and per ET cycle, showing lower pregnancy rate in TAI positive group. Logistic regression models demonstrated TAI positive women have less chance to achieve pregnancy.

According to the results of oocyte number, it could be assumed that thyroid autoantibodies do not affect oocyte, oocyte maturation and its quality, regardless of thyroid autoantibodies presence in FF. Therefore, these results do not support theory that thyroid autoantibodies act on zona pellucida due to molecular mimicry [32], and the interesting fact that ICSI could be a method of choice in TAI positive women because it requires no interaction between the sperm cell and the zona pellucida [14,33]. Other studies showed similar results in fertilization rate, as in our study [34,35], but there are studies showing lower fertilization rate in TAI positive group in comparation to TAI negative group 64.3 *vs* 74.6% (p<0.001) [36], 63 *vs* 72% [14]. According the results considering only embryos, it could be assumed that thyroid autoantibodies do not affect embryo, and that thyroid autoantibodies do not influence preimplantation embryo. On the other hand thyroid antibodies could have an effect on post-implantation embryo as shown in a mice model study where TPOAbs influenced post-implantation embryo development, leading to fetal loss [37].

In the study of Zhong et al., pregnancy rate in group with and without TAI was 33.3 *vs* 46.7%, respectively, and miscarriage rate was 26.9 *vs* 11.8% [36]. Kilic et al., did not confirm that TAI affects oocyte number, number and quality of embryos, fertilization and biochemical pregnancy rate (43.5% TAI *vs* 51.6% control), but confirmed lower clinical pregnancy rate in TAI group (30.4% vs 41.9%) [38]. Risk for miscarriage in TAI positive women is 2 to 4 time higher than in women without TAI [8,10,39–41]. Thangaratinam et al., included 31 studies in meta analysis, to assess the association between thyroid autoantibodies and miscarriage, and showed miscarriage OR 3.90 in cohort studies, and 1.80 in *case-control* studies, and miscarriage relative risk (RR) reduction up to 52% with levothyroxine treatment [42]. Higher risk for early miscarriage is noticed during first trimester [2], when the fetus depends on maternal thyroid hormones. There are studies not confirming effect of TAI on ART outcome [34, 43–46].

In conclusion, thyroid autoantibodies present in FF are not generated in the FF, but cross from the blood.For the first time we showed that concentrations of TSH and fT_4_ in FF are the same in women with and without TAI. TAI does not directly impact oocytes and embryos during ART treatment, but it may have an effect on the post-implantation embryo development. Our results indicate lower ART success rate in women with TAI highlighting the importance of thyroid autoimmunity diagnosis in women presenting with infertility.

## Supporting information

S1 Dataset Data set supporting information file. (XLSX)
